# Possibility to Biotransform Anthracyclines by Peroxidases Produced by *Bjerkandera adusta* CCBAS 930 with Reduction of Geno- and Cytotoxicity and Pro-Oxidative Activity

**DOI:** 10.3390/molecules26020462

**Published:** 2021-01-17

**Authors:** Kamila Rybczyńska-Tkaczyk, Teresa Korniłłowicz-Kowalska, Konrad A. Szychowski

**Affiliations:** 1 Department of Environmental Microbiology, Laboratory of Mycology, The University of Life Sciences, Leszczyńskiego Street 7, 20-069 Lublin, Poland; teresa.kornilowicz@up.lublin.pl; 2 Department of Lifestyle Disorders and Regenerative Medicine, University of Information Technology and Management in Rzeszow, Sucharskiego Street 2, 35-225 Rzeszow, Poland; kszychowski@wsiz.edu.pl

**Keywords:** daunomycin, doxorubicin, versatile peroxidases, genotoxicity, biotoxicity, cytotoxicity

## Abstract

The aim of this study was to evaluate the bioremoval mechanism of anthracycline antibiotics by the white-rot fungus *B. adusta* CCBAS 930. The activity of oxidoreductases and levels of phenolic compounds and free radicals were determined during the biotransformation of anthraquinone antibiotics: daunomycin (DNR) and doxorubicin (DOX) by *B. adusta* strain CCBAS 930. Moreover, phytotoxicity (*Lepidium sativum* L.), ecotoxicity (*Vibrio fischeri*), genotoxicity and cytotoxicity of anthraquinone dyes were evaluated before and after biological treatment. More than 80% and 90% of DNR and DOX were removed by biodegradation (decolorization). Initial solutions of DNR and DOX were characterized by eco-, phyto-, geno- and cytotoxicity. Despite efficient decolorization, secondary metabolites, toxic to bacteria, formed during biotransformation of anthracycline antibiotics in *B. adusta* CCBAS 930 cultures. DNR and DOX metabolites did not increase reactive oxygen species (ROS) production in human fibroblasts and resazurin reduction. DNR metabolites did not change caspase-3 activity.

## 1. Introduction

The development of civilization and accelerated pace of life have led to a global increase in the incidence of cancer in the last decade [1]. Moreover, the increase in the survival age average has increased the occurrence of cancer. This resulted in a more than two-fold increase in the production of anti-tumor drugs [2]. One of the main groups of drugs used to treat cancer are DNA intercalators (cytostatic drugs), which include anthracycline antibiotics (AQA), mainly daunomycin (DNR) and doxorubicin (DOX) [3]. Daunomycin (DNR), also known as daunorubicin, is a chemotherapeutic used to treat cancer, especially acute myeloid and lymphocytic leukemias, chronic myelogenous leukemia and Kaposi’s sarcoma. Doxorubicin (DOX) is used to treat the same cancer as DNR, but additionally, DOX is administered in breast cancer. Currently, China and India (50%), the United States (28%), Europe (15%) and Japan (12%) are the main producers of cytostatic drugs [3].

In recent years, the amount of pharmaceuticals and their metabolites in wastewater, particularly anti-tumor drugs have increased [4]. Depending on the country, the level of anthracycline antibiotics in hospital wastewater and surface water ranges from ng/L to µg/L [4,5,6,7,8,9]. The presence of cytostatic drugs in wastewater and surface water poses a serious threat to the aquatic environment due to their carcinogenic, mutagenic and teratogenic properties [3,10,11,12]. Moreover, modern anthracycline antibiotics are highly resistant to environmental factors. The increased stability of pharmaceuticals impedes their complete degradation during wastewater treatment and allows them to accumulate in the environment. Residues of these drugs can enter surface water and groundwater, endangering both aquatic organisms and human health [13].

Currently, wastewater containing pharmaceuticals is treated using physicochemical methods such as ozonation, adsorption, membrane filtration and electrolysis [3,14]. In recent years, biological methods using fungi to remove pharmaceutical residues in effluents have gained in importance, supplementing or presenting an alternative to conventional methods. Biodegradation is the most frequently discussed biological mechanism of contaminant removal by fungi, and involves exploitation of their natural ability to synthesize extracellular ligninolytic enzymes. Owing to the low substrate specificity of these enzymes, they can be used for biodegradation of anthraquinone derivatives [15,16,17,18,19,20]. The literature lacks information on the degradation mechanism of anthracycline antibiotics by microorganisms. Moreover, we found no information regarding detoxification of anthracycline antibiotics in the aquatic environment by microorganisms. Our previous study has shown that strain CCBAS 930 of *Bjerkandera adusta* is a promising agent in athraquinone derivative biodegradation e.g., anthraquinone dyes, alizarin and anticancer anthracyclines like DNR [19]

Therefore, the aim of this study was to evaluate the mechanism of anthracycline antibiotic bioremoval and detoxification by the white-rot fungus *B. adusta* CCBAS 930. In this study, we characterized the activity of oxidoreductases (i.e., peroxidases, dioxygenases, superoxide dismutase, catalase, glucose oxidase and laccase), levels of free radicals and phenolic compounds during the biotransformation of DNR and DOX. Moreover, bio-, phyto-, geno- and cytoxicity assays were performed to determine the toxicity of its biotransformation products. Considering that anhracyclines are characterized by a strong pro-oxidant capacity, antioxidant properties during their treatment by *B. adusta* CCBAS 930 were assessed.

## 2. Results

### 2.1. AQA Bioremoval by B. adusta CCBAS 930

**Figure 1:**
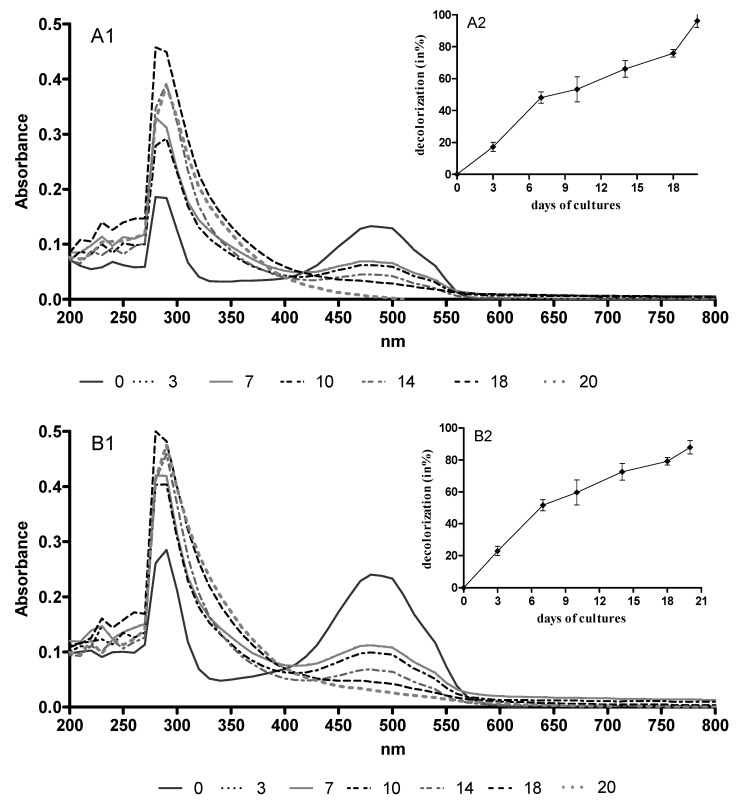
UV-visible spectra (1) and decolorization degree (2) of DNR (**A**) and DOX (**B**) before and during cultures of *B. adusta* CCBAS 930.

We observed decolorization and biosorption of AQAs during treatment with B. adusta CCBAS 930. The highest degree of color removal was observed in the first two weeks. At the end of the second week of *B. adusta* CCBAS cultures with the addition of 10 µg/mL of DNR and DOX, their decolorization ranged from 17.30 to 66.16% and 22.91 to 72.50%, respectively. In 20-day-old cultures, *B. adusta* strain CCBAS 930 removed more than 90% of AQAs. Spectral analysis (200–800 nm) showed that B. adusta CCBAS 930 removed anthracyclines and broke down chromophores, but produced colorless secondary products with maximum absorbance at 216 nm, 271 nm and 364 nm (Figure 1). The degree of DNR and DOX biosorption after B. adusta CCBSA 930 cultures was 13.48% and 7.31%, respectively.

### 2.2. Extracellular Oxidoreductase Activities during AQA Biotransformation in B. adusta CCBAS 930 Cultures

**Figure A1:**
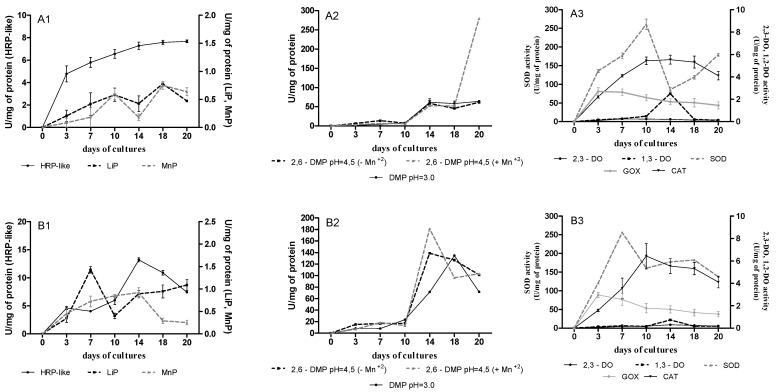
Activities of oxidoreductases (U/mg) in cultures of *B. adusta* CCBAS 930 supplemented with daunomycin (DNR) (**A1**–**A3**) and doxorubicin (DOX) (**B1**–**B3**) (1,2-DO (1,2-dioxygenase), 2,3-DO-(2,3-dioxygenase), CAT (catalase), GOX (glucose oxidase), HRP-like (horsheradish-type peroxidase), LiP (lignin peroxidase), MnP (manganese-dependent peroxidase), PhC (phenolic compounds), SOD (superoxide dismutase), SOR (superoxide radical levels), VP (versatile peroxidases activity measurement without Mn^+2 in pH = 3.0 and pH = 4.5 and with Mn+2 in pH = 4.5).^

The activity of HRP-like, MnP and LiP peroxidases, VP, 2,3 and 1,3 dioxygenases as well as SOD, GOD and CAT was observed in *B. adusta* CCBAS 930 cultures with DNR and DOX; laccase activity was not detected. In case of peroxidases, the highest activity was recorded for VP followed by HRP-like, MnP and LiP. MnP and LiP activities were detected already on the third day in *B. adusta* CCBAS 930 cultures with anthracyclines and they were much lower compared to VP and HRP-like (from 0.10 to 0.90 and from 0.20 to 1.10 U/mg protein, respectively). VP activity, especially measured at pH = 4.5, with and without Mn^+2^, appeared from the first days of *B. adusta* CCBAS 930 cultures with both anthracyclines. VP activities measured at pH = 4.5, without or with Mn^+2^ increased gradually, reaching a maximum of 61–101 and 103–280 U/mg protein on day 20, respectively (Figure A1). In the presence of DOX, the maximum activity of HRP-like, LiP and MnP in *B. adusta* CCBAS 930 cultures was observed at the end of the second week and it was 13.20, 0.94 and 0.92 U/mg protein, respectively.

The activity of GOX, CAT, SOD, 1,2-DO and 2,3-DO was also detected in *B. adusta* CCBAS cultures with anthracyclines. During biotransformation of AQAs by *B. adusta* CCBAS 930, we reported maximum GOX and CAT activities on day 7 (2.56–2.64 U/mg protein) and day 10 (5.44–6.44 U/mg protein), respectively. SOD activity in *B. adusta* CCBAS 930 cultures with DNR and DOX was the highest in the first week (260.56 ± 12.05 and 256.20 ± 10.95 U/mg protein, respectively). Dioxygenase activities were at a lower level compared to other oxidoreductases. In the presence of DNR and DOX, the maximum 2,3-DO and 1,2-DO activities were recorded at the end of the second week of cultures (0.24–0.30 and 0.73–2.50 U/mg protein, respectively) (Figure A1).

### 2.3. Changes of pH during AQA Biotransformation in B. adusta CCBAS 930 Cultures

The pH decreased during the 20-day culture of *B. adusta* strain CCBAS on medium with the addition of DNR and DOX at a concentration of 10 µg/mL. We observed that pH decreased during *B. adusta* CCBAS 930 cultures in the presence of DNR and DOX from pH = 6.0 to 4.09 and 3.90, respectively.

### 2.4. PhC and SOR Contents during AQA Biotransformation in B. adusta CCBAS 930 Cultures

**Figure 2:**
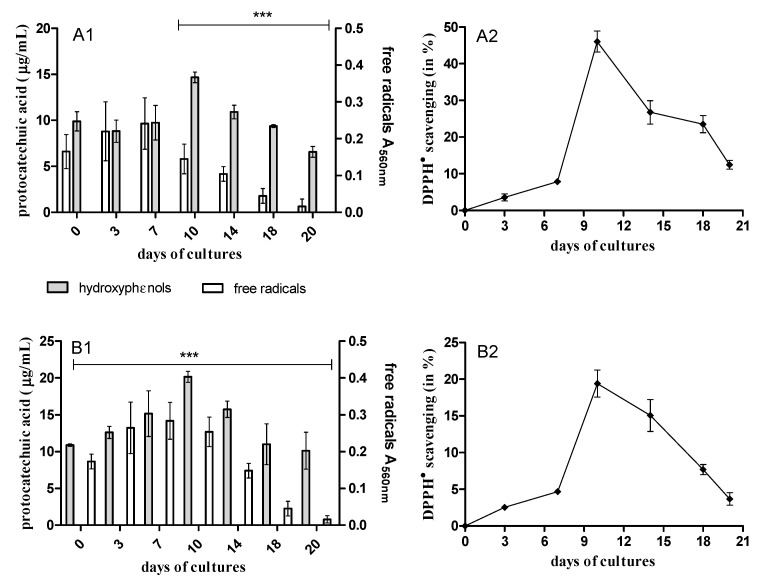
The content of phenols (µg/mL of protocatechuic acid), level of free radicals (A_560nm_) (1) and antioxidants properties (2) during treatment of DNR (**A**) or DOX (**B**) by *B. adusta* CCBAS 930 *** *p* < 0.001.

During the first 10 days of *B. adusta* CCBAS 930 cultures with DNR and DOX, a systematic increase in the content of phenolic compounds and free radicals was observed. In the third week, the content of phenolic compounds in *B. adusta* CCBAS 930 cultures with the addition of DNR and DOX decreased to the value characteristic for the initial AQA solutions (Figure 2A1,B1). We did not observe a reduction in phenolic compound concentrations compared to AQA control solutions before *B. adusta* CCBAS 930 treatment. However, a systematic decrease in the content of free radicals was observed in *B. adusta* CCBAS 930 cultures with the addition of DNR and DOX (90.30% and 90.70%) (Figure 2A1,B1).

### 2.5. Evaluation of Antioxidative Activity of AQAs during Biological Treatment

We observed that initial compounds and their biotransformation products were characterized by different anti-/prooxidative properties during *B. adusta* CCBAS 930 cultures with DNR and DOX. Initial DNR and DOX solutions (10 µg/mL) in the DPPH scavenging assay did not show any antioxidant activity; on the contrary, they showed strong oxidative properties towards trolox (data not shown). Antioxidant activity of post-liquid cultures systematically increased during *B. adusta* CCBAS 930 cultures with maximum DPPH scavenging activity to the 2nd week (20–46%). After that time, a decrease in their antioxidant activity was recorded (Figure 2A2,B2).

### 2.6. Comparative Evaluation of AQA Toxicity before and after B. adusta CCBAS 930 Treatment

#### 2.6.1. Phytotoxicity

**Figure 3:**
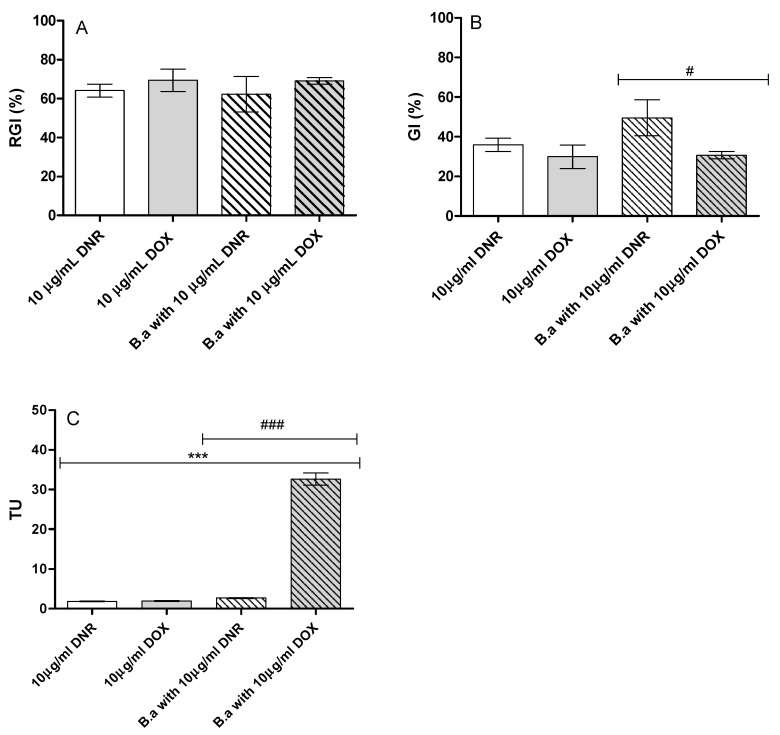
Phyto- (**A**,**B**) and biotoxicity (**C**) initial solutions of DNR and DOX (10 µg/mL) and decolorized post-culture liquids of *B. adusta* CCBAS 930 (RGI—root growth inhibition, GI—germination index, TU- Toxicity Units: *** *p* < 0.001 versus the control anthracycline antibiotics: DNR and DOX, ### *p* < 0.001; # *p* < 0.05 between post-cultured fluids *B. adusta* CCBAS 930.

Root growth inhibition (RGI) was at a comparable level (62–69%) for anthracyclines before and after *B. adusta* CCBAS 930 treatment, with no apparent differences (Figure 3A). Significant differences (*p* < 0.05) were found in the inhibition of *L. sativum* seed germination between post-culture fluids after DNR and DOX treatments (GI = 49.55 ± 9.05 and 30.70 ± 1.80, respectively). Decolorized post-culture fluids with DNR or DOX were characterized by slightly higher, but not statistically significantly different, germination index (GI) than the initial 10 µg/mL DNR and DOX solutions (GI = 35.85 ± 3.33 and 29.88 and 22.06 ± 5.94) (Figure 3B).

#### 2.6.2. Ecotoxicity

The ecotoxicity assay with *V. fischeri* bacteria (Microtox) showed that initial media with the addition of DNR and DOX at a concentration of 10 µg/mL were characterized by acute toxicity (TU = 1.79 ± 0.09 and TU = 1.90 ± 0.13, respectively). Surprisingly, we found significant differences in the toxicity of decolorized post-culture fluids with anthracycline antibiotics. Post-culture fluid with DNR had a similar level of toxicity as the initial 10 µg/mL DNR solution, but in the case of post-culture fluids with DOX, we observed several times higher toxicity (high acute toxicity) against *V. fischeri* compared to the initial media with the addition of 10 µg/mL DOX (TU = 2.67 ± 0.13 and TU = 31.22 ± 1.53, respectively) (Figure 3C).

#### 2.6.3. Genotoxicity

Genotoxicity of anthracyclines before and after treatment by *B. adusta* CCBAS 930 was also investigated. Our results clearly showed genotoxicity of the initial 10 µg/mL DNR and DOX solutions. The effects were dose-dependent and decreased with increasing sample dilutions. The highest values of CIF factor was noted for DNR and DOX initial solutions at a concentration of 10 µg/mL (CIF = 13.42 and 9.58, respectively) (Table A1). After biological treatment of DNR and DOX by *B. adusta* CCBAS 930, genotoxicity of 20-day post-liquid cultures was on average 95% and 90% lower, respectively. For all tested samples, we did not observe any genotoxicity with metabolic activation (with S9 fraction; data not shown).

#### 2.6.4. Cytotoxicity

**Figure A2:**
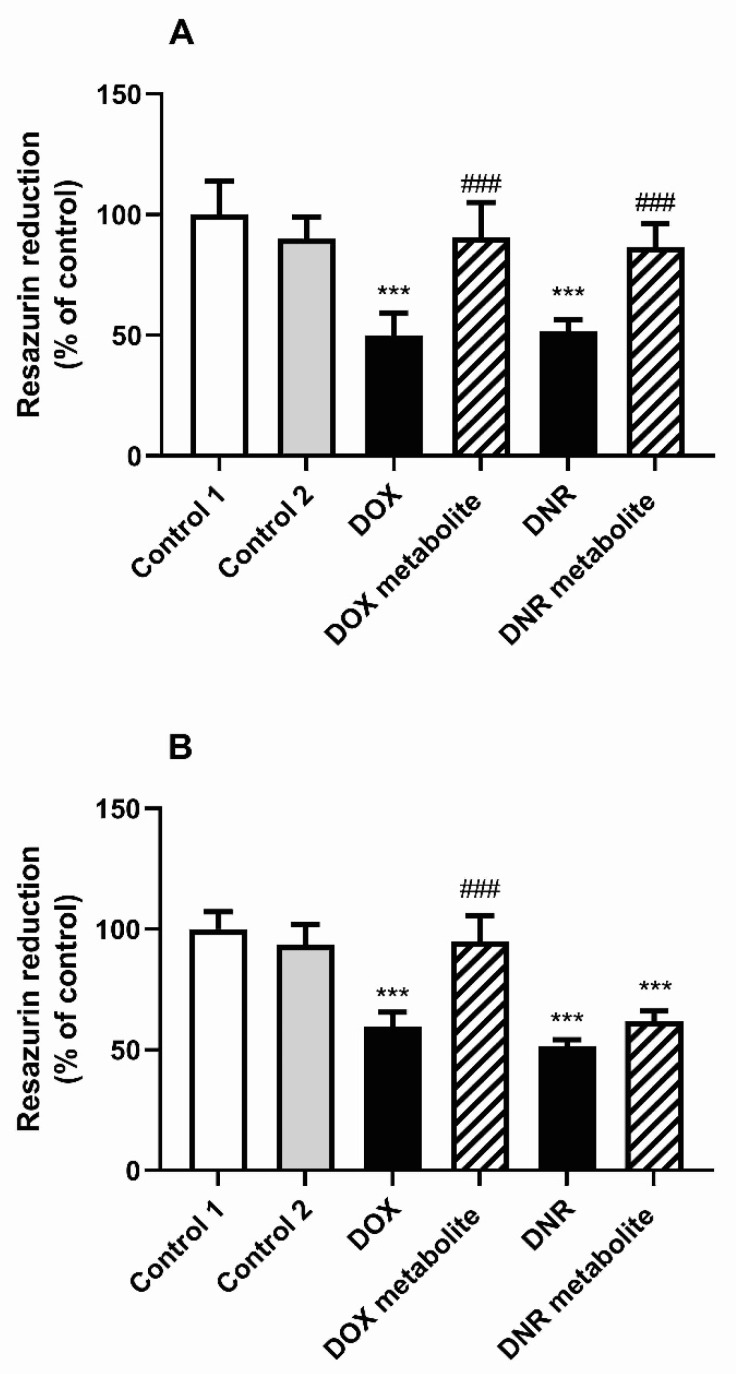
The effect of conditioned media on the resazurin redution after 24 h (**A**), and 48 h (**B**) in BJ fibroblasts: *** *p* < 0.001 versus the control 1 cultures. ### *p* < 0.001 versus the cell espoused to medium before conditioning. Control 1—cell culture medium with PBS, Control 2—cell culture medium with Park Robinson medium for fungi culture, DOX—cell culture medium with doxorubicin, DOX metabolite—cell culture medium with doxorubicin after fungi culture, DNR—cell culture medium with daunomycin, DNR metabolite—cell culture medium with daunomycin after fungi culture.

BJ cell exposure to initial DOX and DNR solutions in medium cause a decrease in resazurin reduction by 50.36 and 48.49% after 24 h, respectively, and 40.36 and 48.40% after 48 h, respectively, compared to Control 1. Conditioned media containing DOX and DNR metabolite did not affect BJ cell metabolism after 24 h compared to Control 1. Only media containing DOX metabolite did not affect BJ cell metabolism after 48 h, as measured by the resazurin reduction assay compared to Control 1 (Figure A2).

**Figure 4:**
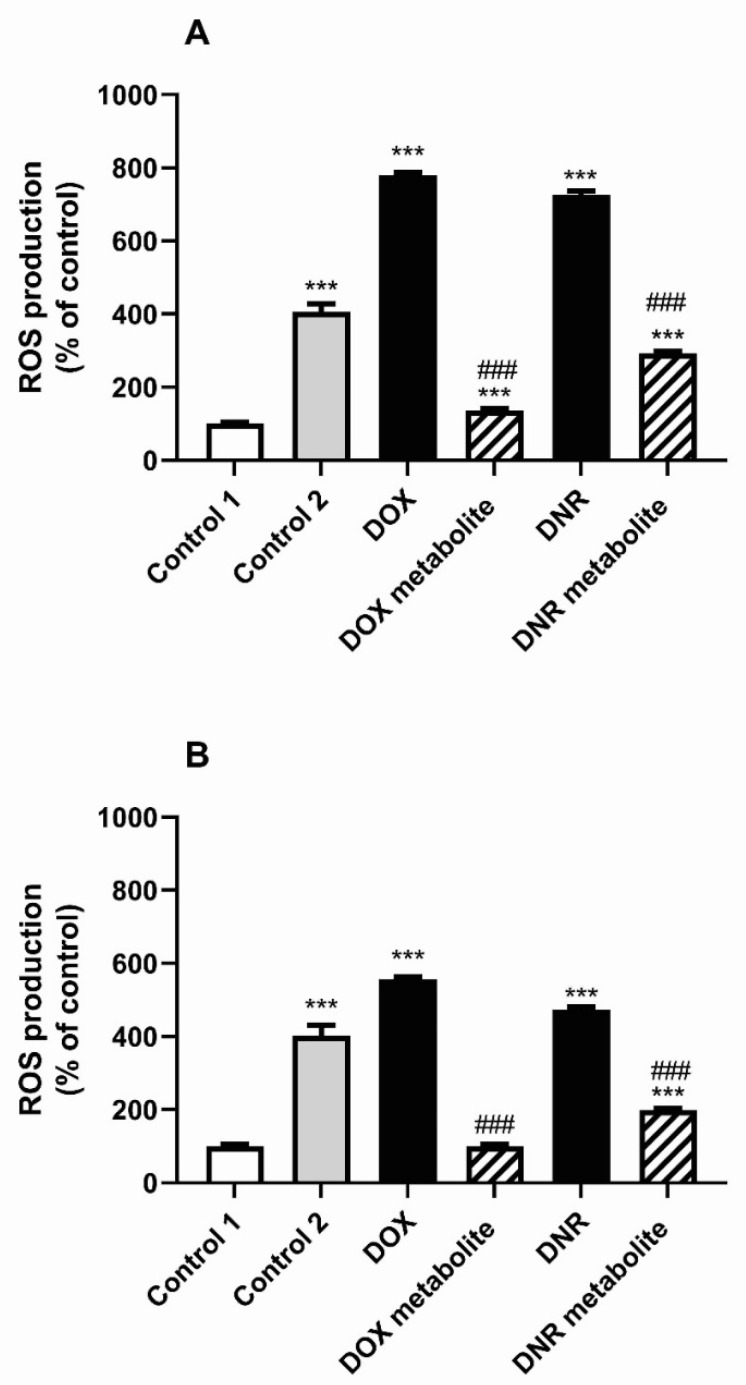
The effect of conditioned media on the ROS production after 24 h (**A**), and 48 h (**B**) in BJ fibroblasts: *** *p* < 0.001 versus the control 1 cultures. ### *p* < 0.001 versus the cell espoused to medium before conditioning. Control 1—cell culture medium with PBS, Control 2—cell culture medium with Park Robinson medium for fungi culture, DOX—cell culture medium with doxorubicin, DOX metabolite—cell culture medium with doxorubicin after fungi culture, DNR—cell culture medium with daunomycin, DNR metabolite—cell culture medium with daunomycin after fungi culture.

After 24- and 48-h exposure of BJ cells to the medium containing initial DOX and DNR solutions, ROS production was induced by 680.22 and 625.72%, respectively, after 24 h and 457.34 and 372.97%, respectively, after 48 h compared to Control 1. Interestingly, Control 2 fungal medium showed an increase in ROS production by 306.48 and 302.67%, respectively, compared to Control 1 at both tested time intervals. After 24 and 48 h of BJ cell exposure to conditioned medium containing DOX metabolite and DNR metabolite, ROS production decreased compared to the medium containing DOX and DNR (a decrease by 642.77 and 434.05% after 24 h, respectively, and 457.21 and 273.36% after 48 h, respectively) (Figure 4).

**Figure 5:**
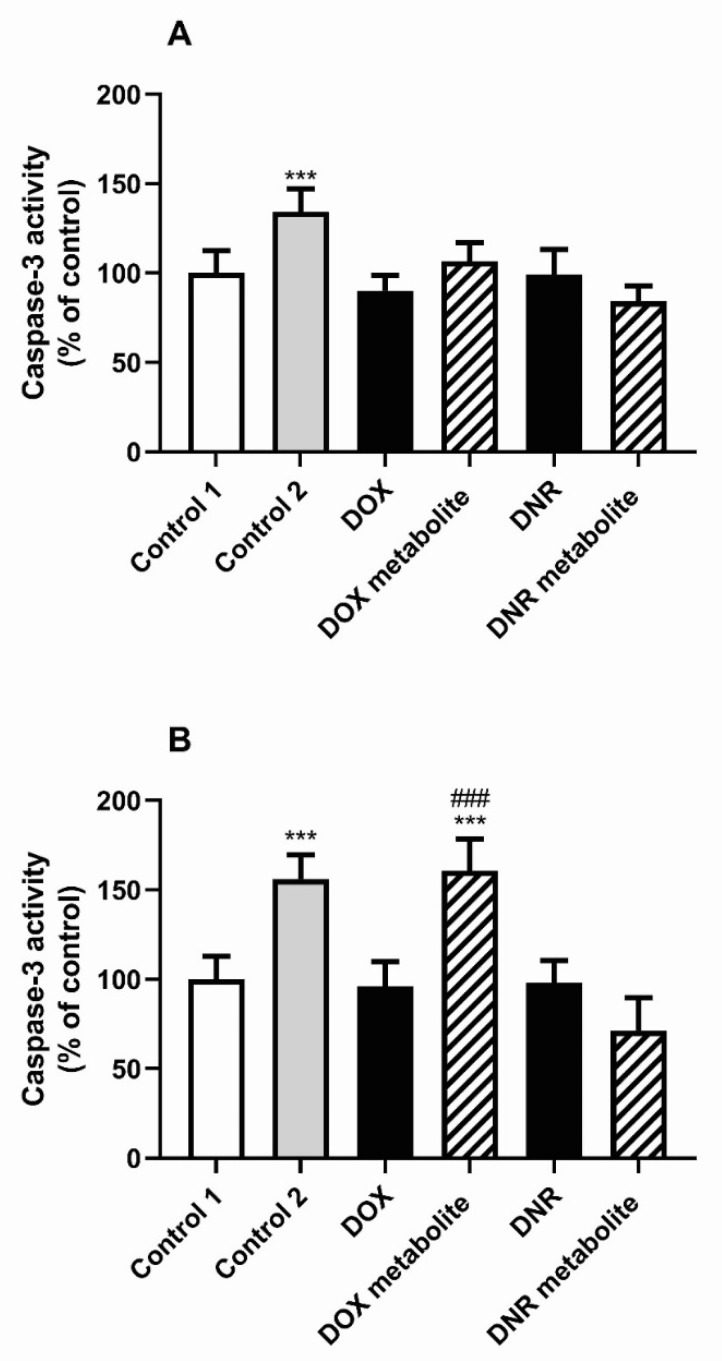
The effect of conditioned media on the caspase-3 activity after 24 h (**A**), and 48 h (**B**) in BJ fibroblasts: *** *p* < 0.001 versus the control 1 cultures. ### *p* < 0.001 versus the cell espoused to medium before conditioning. Control 1—cell culture medium with PBS, Control 2—cell culture medium with with Park Robinson medium for fungi culture, DOX—cell culture medium with doxorubicin, DOX metabolite—cell culture medium with doxorubicin after fungi culture, DNR—cell culture medium with daunomycin, DNR metabolite—cell culture medium with daunomycin after fungi culture.

Caspase-3 activity was not affected after 24 h of BJ cell exposure to the medium containing initial DOX and DNR solutions. Interestingly, caspase-3 activity increased by 34.00 and 56.00%, respectively, at both studied time intervals (24 and 48 h) in the group containing 10% fungal mineral Park Robinson medium (Control 2) compared to Control 1 cells containing 10% PBS. After 48 h, DOX metabolite increased caspase-3 activity compared to Control 1 and DOX-containing group (by 60.54% compared to Control 1) (Figure 5).

**Figure A3:**
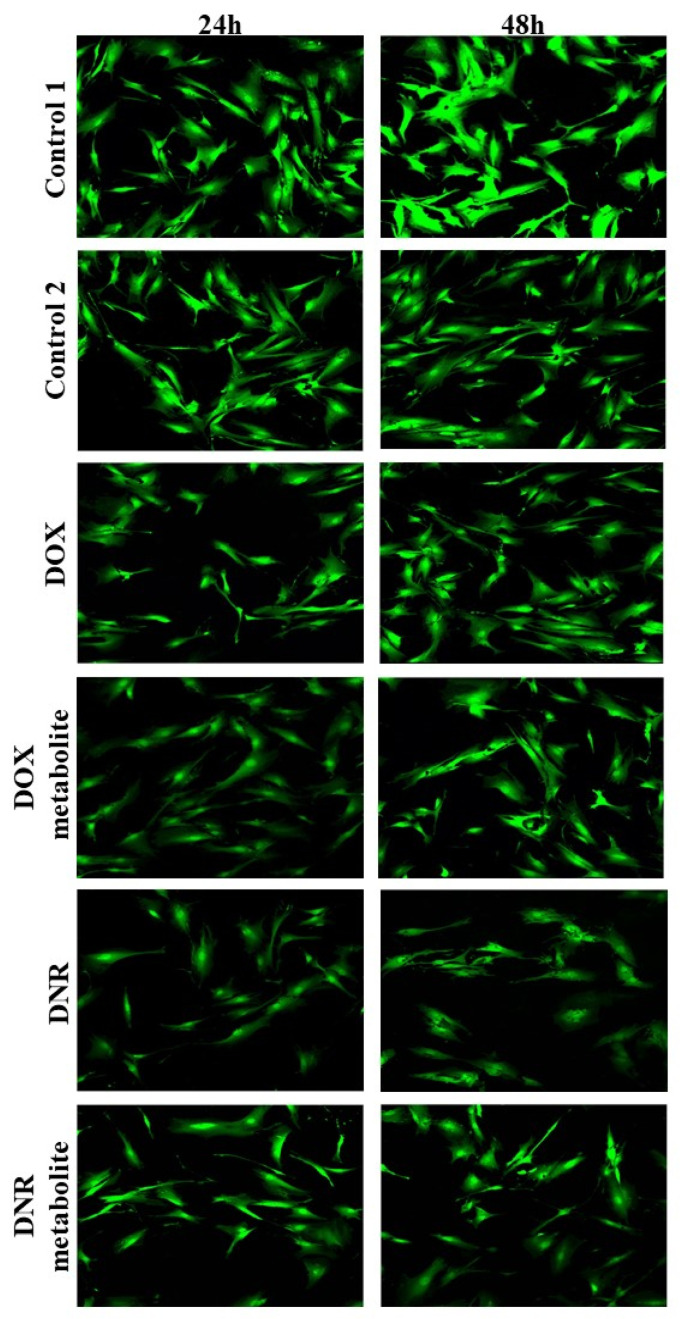
The effect of conditioned media on the calcein AM after 24 h, and 48 h in BJ fibroblasts. Control 1—cell culture medium with PBS, Control 2—cell culture medium with Park Robinson medium for fungi culture, DOX—cell culture medium with doxorubicin, DOX metabolite—cell culture medium with doxorubicin after fungi culture, DNR—cell culture medium with daunomycin, DNR metabolite—cell culture medium with daunomycin after fungi culture. Photomicrographsare shown at 200× magnification.

Viable cells in this assay had a light green fluorescent cytoplasm. Healthy cells with green fluorescent cytoplasm were predominant in the control cultures. After 24 and 48 h, we observed a decrease in cell number in the group treated with DOX and DNR compared to Control 1. We observed an increase in BJ cell number at both time intervals in the cell group exposed to DOX metabolite and DNR metabolite compared to cells exposed to DOX and DNR (Figure A3).

### 2.7. Determination of Main Principal Components (PC) during AQA Biotransformation in B. adusta CCBAS 930 Cultures

**Figure A4:**
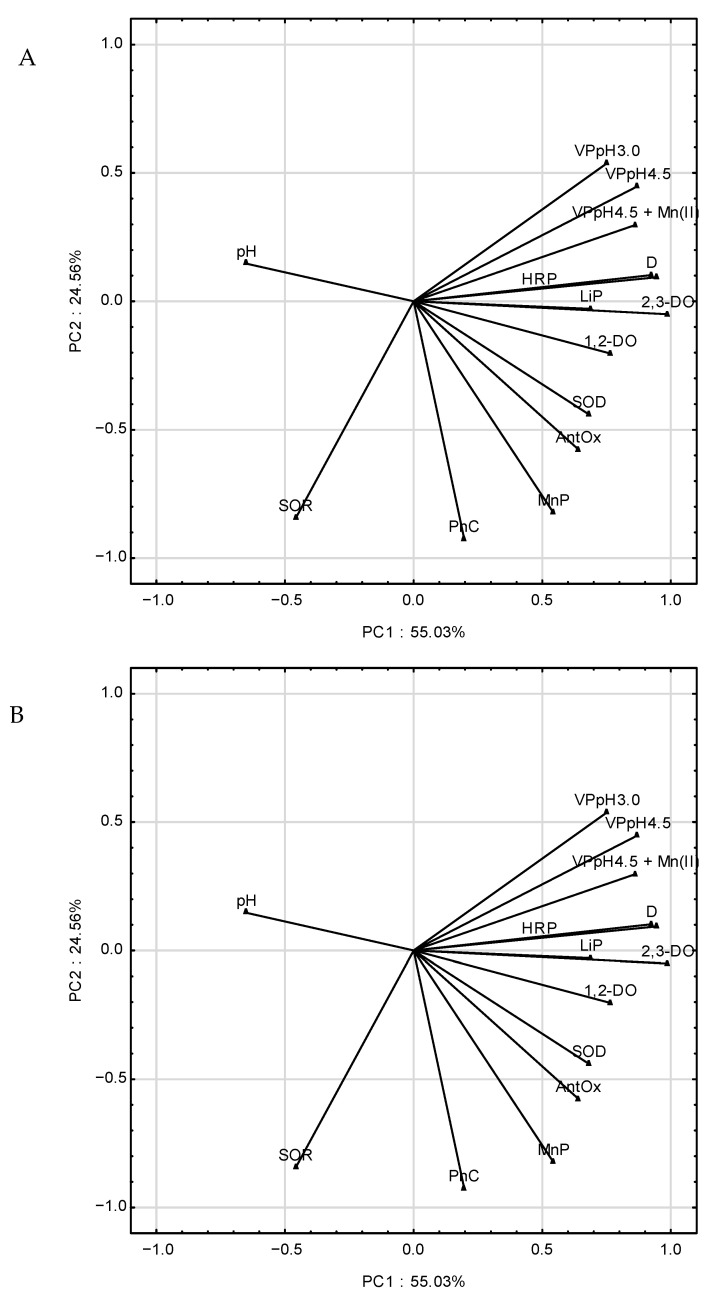
Plot of variables. Location of load vectors towards two principal components for cultures of strain of *B. adusta* CCBAS 930 with DNR (**A**) and DOX (**B**); 1,2-DO (1,2-dioxygenase), 2,3-DO—(2,3-dioxygenase), CAT (catalase), D—decolorization, GOX (glucose oxidase), HRP-like (horsheradish-type peroxidase), LiP (lignin peroxidase), MnP (manganese-dependent peroxidase), PhC (phenolic compounds), SOD (superoxide dismutase), SOR (superoxide radical levels), VP (versatile peroxidases activity measurement without Mn^+2 in pH = 3.0 and pH = 4,5 and with Mn+2^ in pH = 4.5).

Two principal components, PC1 and PC2, were found to be associated with DNR and DOX decolorization by *B. adusta* CCBAS 930 and they explained 79.98–81.53% of data variability. PC1 explained 54.99 and 55.22% of data variability in *B. adusta* CCBAS 930 cultures with DNR and DOX, respectively. In both cases, PC1 was mainly associated with negatively correlated (*p* < 0.05) SOR (74.50–87.72%) and GOX (92.76–90.33%) activities and pH (77.06–79.58%) and positively correlated with VP peroxidase activity, measured at pH = 3.0 and pH = 4.5 (with/without Mn^+2^) (88.22–95.78%). In *B. adusta* CCBAS 930 cultures with DOX, PC1 was strongly associated with HRP-like (90.30%) and 2,3-DO activity (90.60%). In *B. adusta* CCBAS 930 cultures with DNR and DOX, PC2 factor explained 24.99% and 26.30% of anthracycline biotransformations, respectively. In *B. adusta* CCBAS930 cultures with AQAs, PC2 was strongly associated with a higher content of phenolic compounds (73.38–97.48%) and antioxidant properties (83.50–95.80%) for DNR and LiP (74.72%) and MnP (94.61%) activity for DOX. PC2 for DOX treatment in *B. adusta* CCBAS 930 cultures was associated with higher activities of CAT (86.60%), SOD (72.23%) and 2.3-DO (81.26%) (Figure A4).

## 3. Discussion

Due to the increase in cancer incidence, cytostatics are one of the main environmental problems. Their main sources are hospitals, municipal and industrial wastewater containing non-metabolized or partially metabolized cytostatics and effluents from their production. Since these compounds are not efficiently removed during wastewater treatment, they are found in ground, surface and even drinking water. There is no information in the literature on the biotransformation of anthracycline antibiotics by fungi. Our team was the first to suggest the possibility of using the *B. adusta* strain CCBAS 930 to remove these pharmaceuticals from the aquatic environment [19]. Biotransformation through enzymatic decolorization and biosorption was observed during biological treatment of anthracycline antibiotics with *B. adusta* CCBAS 930. However, the main mechanism of DNR and DOX removal (more than 80 and 90%, respectively) was associated with color removal through enzymatic oxidation. Previous data indicated the connection between color removal (UV-VIS spectrum analysis) and biodegradation of color compounds, e.g., synthetic dyes. Color removal of these compounds was visible when the chromophoric center of the dye was cleaved [20,21]. During *B. adusta* CCBAS 930 cultures with AQAs, we observed a systematic color decrease at an absorbance of 480 nm, but it was slightly increased when oxidized products were formed. Reszka et al. (2003) [22] showed increased absorbance at 216 nm, 271 nm and 364 nm during DNR oxidation by peroxidases, implying that the observed spectral changes were connected with DNR chromophore conversion into its non-colored oxidation products.

During biotransformation of DNR and DOX, *B. adusta* CCBAS 930 synthesized the following peroxidases: HRP-like, MnP, LiP and VP, but no laccase. Previous data demonstrated that the main oxidoreductases responsible for decolorization and biodegradation of anthraquinone derivatives, e.g., anthraquinone dyes in *Bjerkandera* sp. were peroxidases [16,17,23]. Our study indicated that the removal of AQAs in *B. adusta* CCBAS cultures was related to their biodegradation by extracellular peroxidases, especially VP. Reszka et al. (2003, 2005) [22,24] described oxidation mechanism of anthracyclines based on peroxidases. The latter authors suggested that one possible mechanism of anthracycline oxidization could be via a reaction catalyzed by peroxidases in the presents of H_2_O_2_ [24]. We reported Mn-dependent peroxidase oxidization of phenolic compounds, but also their oxidization to Mn(III) at the Mn(II) binding site, which indicated that VP peroxidase synthesis had a wide substrate range. Our previous study indicated that VP oxidized not only phenolic compounds but also high-redox potential substrates e.g., anthraquinone dyes Alizarin Blu Black B and Acid Blue 129 [23]. Moreover, VP had a high application value due to the effective degradation of PHAs [25], pharmaceuticals and hormones [26] as well as endocrine disrupting compounds, e.g., Bisphenol A and Triclosan [27,28]. An important parameter that affects not only enzyme’s activity but also its affinity to the substrate is pH. During biotransformation of AQAs by *B. adusta CCBAS*, we observed pH decrease, which was strongly correlated (*p* < 0.05) with increased activities of VP peroxidases. The connection between VP activity, Mn^+2^ oxidation and guaiacol without and in the presence of Mn^+2^ and medium pH has been confirmed previously [29].

We observed high phenolic compound production during *B. adusta* CCBAS 930 cultures with anthracycline antibiotics. However, our results indicated that this phenolic compounds showed antioxidant activity, e.g., scavenging activity towards DPPH^•^ radicals. Earlier study indicated that phenolic compounds could be produced during the growth of different white-rot fungi or during xenobiotic biotransformations, e.g., anthraquinone derivatives through the activity of fungal oxidoreductases [23]. We also showed phenolic compounds in the control culture of *B. adusta* CCBAS 930 without the addition of anthracycline antibiotics (data not shown). Moreover, previous data have demonstrated that phenols are substrates for dioxygenases that catalyze the cleavage of aromatic rings [30,31]. It is consistent with our data, which showed a correlation (*p* < 0.05) between an increase in hydroxyphenol concentrations and 2,3-DO activity during AQA biotransformation. We observed SOR production during AQA treatment by *B. adusta* CCBAS 930, but also in control cultures without AQAs (data not shown). This indicated that partial oxygen reduction, with the release of H_2_O_2_ molecule was the main source of O^•-^ in fungal cells during the biotransformation of AQAs. It is worth mentioning that free radicals are formed not only due to oxidative stress, but also physiological changes. Even high ROS levels do not adversely affect the growth and development of these fungi as they have an enzymatic mechanism that neutralizes ROS such as SOD, CAT and peroxidase [32]. Biosynthesis mechanism of extracellular peroxidases in white-rot fungal cultures is related to glucose metabolism, resulting in ROS formation [32]. This was confirmed by the correlation (*p* < 0.05) between the increase in the level of free radicals during the cultivation of *B. adusta* CCBAS 930 and higher glucose oxidase activity. H_2_O_2_ is then broken down to O_2_ and H_2_O by catalase or participates in peroxidase-catalyzed reactions as a co-substrate. Reszka et al. (2005) [24] showed that HRP peroxidase could oxidize hydroquinone in an anthracycline molecule, thereby generating free radicals (phenoxyl radical) and causing its degradation. On the other hand, SOD is a key enzyme responsible for O^•-^ removal during the degradation of high redox potential compounds, e.g., ligninocelulose and anthraquinone derivatives [23,33].

However, xenobiotics such as pharmaceuticals in the environment may contribute to the formation of free radicals. Anthracycline antibiotics readily undergo single-electron reduction, generating semiquinone radicals and superoxide anion radicals. This initiates a cascade of free radical reactions whose products are highly toxic hydrogen peroxide and hydroxyl radical. Due to the universal character of free-radical mechanism of action of anthracyclines, all cells are exposed to harmful effects of radicals generated [13]. In light of this information, it can be assumed that not only oxydoreductases, but also phenolic compounds, formed during the biotransformation of anthraquinone derivatives, are responsible for the detoxification of these compounds by free radical neutralization, especially since we also showed a decrease in free radicals during removal of AQAs in *B*. *adusta* CCBAS930 cultures.

In addition to decolorization degree and activity of oxidoreductases, we also assessed bio-, phyto-, cyto- and genotoxicity of DNR and DOX before and after *B. adusta* CCBAS 930 treatment. Using an ecotoxicology assay with *Vibrio fischeri*, we did not show a reduced toxicity of post-culture liquids obtained after treating 10 µg mL^−1^ DNR and DOX with *B. adusta* CCBAS 930. Post-liquid cultures were characterized by a higher toxicity after treatment with *B. adusta* CCBAS 930. In recent years, various information has been published on the possibility of detoxification of anthraquinone derivatives, e.g., textile dyes by oxidoreductases synthesized by white-rot fungi [23,34,35]. However, the authors’ opinions are ambiguous, as some publications showed an increase in the toxicity of post-culture fluids after biotransformation [36,37]. Based on our results, we suggested that high biotoxicity level after anthracycline treatments with *B. adusta* CCBAS 930 was probably connected with a high concentration of phenolic compounds. On the other hand, we observed that the modification of *B. adusta* CCBAS 930 culture could significantly reduce the biotoxicity of anthracyclines (data not shown). Previous study indicated that phenolic compounds produced by white-rot fungi exhibited antibacterial activity [38,39]. Culture fluids of white-rot fungi such as *Lentinula edodes* and *Pleurotus ostreatus* were characterized by antibacterial activity towards Gram-positive and Gram-negative bacteria [38,39]. Moreover, anthraquinone derivatives, e.g., natural dyes and those used in the textile industry have antibacterial properties. The mechanism of their antibacterial activity is primarily based on blocking cell wall biosynthesis, disrupting transport through the cytoplasmic membrane and inhibiting the synthesis of nucleic acids. In addition, exposing bacteria for a long period of time to anthraquinones caused that this compounds entered and accumulated in the cells, which resulted in their higher toxicity [40]. In view of recent studies, the use of *V. fischeri* (Microtox) as the main assay to determine the ecotoxicity potential of anthraquinone derivatives in culture fluids after treatment with microfungi cannot be considered as one of the most important toxicological tests.

Phytotoxicity of post-culture fluids of *B. adusta* CCBAS 930 was determined based on the inhibition of seed germination and root elongation. Seed germination and root growth inhibition of *L. sativum* L. in the presence of initial DNR and DOX solutions or post-culture liquids were at the same levels. There is some information in the literature, on the use of the phytotoxicity assay to determine the detoxification of anthraquinone derivatives by fungi [20,23]. However, the authors reported an increase of anthraquinone derivatives e.g., textile dyes after fungal treatment [20] and a decrease of their phytotoxicity [23]. Our study showed no increase in phytotoxicity after AQA treatment by *B. adusta* CCBAS 930. Due to the fact that the deficit of drinking water increases with climate change, work is still ongoing on effective methods of municipal and industrial wastewater recycling that will allow for their re-use, e.g., for agricultural irrigation [41]. Our results are very important from the point of view of the potential application of the fungus *B. adusta* CCBAS 930 for removal of these cytostatic drugs and re-use of the post-culture fluids, e.g., for agricultural irrigation.

Genotoxicity is also a very important issue in the effective removal of pollutants using biological methods. Genotoxicity and potential promutagenic effects of xenobiotics, e.g., cytostatic drugs, have serious consequences in the future. Previous data indicated that certain amounts of cytostatic drugs pass unmetabolized through the patient. Moreover, cytostatic agents and their metabolites are continuously disposed of not only in hospitals, but also municipal wastewater and end up in the wastewater system [4,42]. Zhang et al. (2013) [3] reported that metabolic products of anthracycline antibiotics excreted with urine still exhibited carcinogenic properties. Our study indicated high genotoxicity of initial DNR and DOX solutions. After *B. adusta CCBAS* 930 treatment both AQAs showed no genotoxicity effect in the SOS Chromotest assay, without and with metabolic activation. A study by Zounková et al. (2007) [43] showed that doxorubicin exhibited genotoxic properties, as measured by the SOS Chromotest, without and with metabolic activation, at a concentration of 0.074 and 0.098 mg/L, respectively. Our study did not show any genotoxic effects, thus it can be assumed that the biological method of AQA removal from wastewater using *B. adusta* strain CCBAS 930 may be an alternative or supplement to conventional pharmaceutical wastewater treatment methods.

Additionally, the cytotoxicity assay was carried out before and after fungal treatment. Initial DNR and DOX solutions were also toxic to the BJ skin fibroblast cell line. Control media did not increase ROS production, while media with DNR and DOX strongly enhanced ROS production in BJ skin fibroblasts. The obtained data suggested that both DNR and DNR media caused apoptosis with increasing ROS production, but DNR and DOX metabolites were characterized by low ROS levels and did not activate apoptosis. Previous data demonstrated that high ROS production could lead to the activation of apoptotic processes through the mitochondrial pathway [44]. Low ROS concentration is essential to maintain redox balance and stimulate cell proliferation, but a high level of accumulated ROS may cause cell damage by oxidation of proteins, lipids and DNA, and is indicative of apoptosis [45]. Recent study demonstrated that organic compounds and polyphenols were responsible for ROS generation in cancer cells, but in normal cells they scavenged ROS [45]. Jabłońska-Trypuć et al. (2018) [45] indicated that ROS level in fibroblast cells pretreated with cichoric acid before DOX addition was significantly lower than without cichoric acid treatment. This was in line with our research, which showed that increasing concentrations of hydroxyphenols in culture medium were correlated with their high antioxidant activity. Our study found that the activity of general apoptotic marker such as caspase-3 remained unchanged in post liquid cultures after *B. adusta* CCBAS 930 treatment in group contained DNR. Our results are comparable to a similar assay involving fungal treatment of anthraquinone derivatives, e.g., textile dyes [23,35]. No cytotoxicity effect was observed in fibroblast BJ cells after Alizarine Blue Black B and Acid Blue 29 treatment with *B. adusta* CCBAS 930 [23]. Vanhulle et al. (2008) [35] demonstrated in a cytotoxicity assay with the Caco-2 cell line that the fungus *P. sanguineus* did not produce toxic metabolites during dye-containing wastewater treatments. Moreover, our study indicated that the high antioxidant activity was correlated with decrease in SOR levels. This suggested that biotransformation products such as phenols can protect fibroblast cells by ROS during the biological treatment of anthracyclines in *B. adusta* CCBAS 930 cultures.

## 4. Materials and Methods

### 4.1. Chemicals

Daunomycin hydrochlorine (≥90%), doxorubicin hydrochlorine (≥98%), streptomycin (≥98%), phosphate-buffered saline (PBS) without Ca^2+^ and Mg^2+^, fetal bovine serum (FBS), resazurin, MEM medium without phenol red (≥98%), o-dianisidine (≥95%), veratryl alcohol (96%), 2,6-dimethoxyphenol (99%), ABTS (2,2′-azino-bis(3-ethylbenzothiazoline-6-sulphonic acid) (≥98%), DPPH^•^ (2,2-diphenyl-1-picrylhydrazyl), trolox (97%), peroxidase, type II from horseradish (200 KU) and catechol (≥99%) were purchased from Sigma-Aldrich (St. Louis, MO, USA). All other chemicals and reagents were of analytical grade.

### 4.2. Cultures of Bjerkandera adusta CCBAS 930

The anamorphic *B. adusta* strain CCBAS 930 was isolated from black earth soil (Pheozems, FAO) from a field near Lublin in south-eastern Poland [16]. The experiments were conducted in 100 mL of liquid mineral medium supplemented with DNR or DOX at a concentration of 10 µg/mL according to the method described by Rybczyńska-Tkaczyk et al. (2020) [23]. Control treatment consisted of cultures without the addition of anthracyclines and non-inoculated medium.

### 4.3. Bioremoval of Anthracyclines by B. adusta CCBAS 930

To explain the bioremoval mechanism of anthracycline antibiotics we applied two parameters: decolorization and biosorption degree. Decolorization was estimated by periodic absorbance (A_480nm_) measurements of clear post-culturing liquids with DNR and DOX [16]. Moreover, a visible spectrum in the wavelength range from 200 nm to 800 nm was measured during treatment of anthracyclines in *B. adusta* CCBAS 930 cultures. The biosorption assay was performed on 20-day *B. adusta* CCBAS 930 cultures according to Rybczyńska-Tkaczyk and Korniłowicz-Kowalska (2016) [46]. After cultures (21 days), the mycelia were separated from the media, rinsed 3 times in sterile water and transferred to 50 cm^3^ of 70% ethanol to determine the sorption capacity of the biomass and agitated for 24 h (130 rpm, 28 °C). The sorption capacity was estimated according to the equation:

(1) $$Qe=\frac{(Ci-Cf)V}{m}$$

where *Qe*—biosorption capacity (mg g^−1^), *Ci* and *Cf*—initial and final concentration (mg L^−1^), *m*—adsorbent dose (g), *V*—volume of solution (l).

### 4.4. Evaluation of Oxidoreductase Activities

The activities of the following extracellular oxidoreductases: horseradish-type peroxidase (HRP-like), manganese-dependent peroxidase (MnP), lignin peroxidase (LiP), versatile peroxidase (VP) Mn-dependent and Mn-independent activity, laccase (Lac), 2,3-dioxygenase (2,3-DO), 2,3-dioxygenase (2,3-DO) and superoxide dismutase (SOD) were evaluated using the microplate assay [23]. The activity of horseradish-type peroxidase (HRP-like) and laccase (Lac) was assayed using 255 µL of 0.01% o-dianisidine (ε_460nm_ = 11,3 M^−1^ cm^−1^) in 0.1 M acetate buffer (pH 5.5) with 0.1 mM H_2_O_2_ and 0.5 mM ABTS (ε_420nm_ = 36,000 M^−1^ cm^−1^) as a substrate and 35 µL, respectively. The activity of manganese-dependent peroxidase (MnP) was determined by oxidation of 15 µL of 1 mM MnSO_4_ in 265 µL of sodium malonate (50 mM, pH 4.5) in the presence of 50 µL of the supernatant and 10 µL of 6 mM H_2_O_2_, and subsequent determination of the Mn^+3^–malonic acid complex (ε_270nm_ = 11.590 M^−1^ cm^−1^). The activity of lignin peroxidase (LiP) was assayed using 20 mM veratryl alcohol (ε_310nm_ = 9.300 M^−1^ cm^−1^) in 40 mM tartrate buffer, pH 3, in the presence of 10 µL of 8 mM H_2_O_2_. Versatile peroxidase (VP) activity was determined by the oxidation of 20 mM 2,6-dimethoxyphenols (2,6-DMP). Mn-independent activity of VP was estimated by the oxidation of 15 µL of 20 mM 2,6-DMP (*ε*_468_ = 49.6 M^−1^ cm^−1^) in 265 µL 50 mM sodium malonate buffer (pH 3.0 and 4.5) with 10 µL of 6 mM H_2_O_2_. Mn-dependent activity of VP was assayed by oxidation of 15 µL 20 mM 2,6-DMP in 250 µL 50 mM sodium malonate buffer (pH 4.5) in the presence of 10 µL of 6 mM H_2_O_2_ and 15 µL 0.1 mM MnSO_4._ The activity of 1,2-dioxygenase (1,2-DO) was determined by oxidation of catechol (220 μL, 50 mM) in phosphate buffer pH 7.5 (50 mM) with the presence of 20 μL of Na_2_EDTA (20 mM) and 60 μL of supernatant. The activity of 2,3-dioxygenase (2,3-DO) was estimated by oxidation of catechol (240 μL, 50 mM) in phosphate buffer, pH 7.5 (50 mM), and 60 μL of the supernatant. The activities of catalase (CAT) and glucose oxidase (GOX) were estimated according to Korniłłowicz-Kowalska and Rybczyńska (2015) [47]. Activity of GOX was estimated in glycerol triphosphate buffer (pH 7.0) with o-dianisidine and horseradish peroxidase (ε_525nm_ = 1,86 M^−1^ cm^−1^). Activity of CAT was measurement in the presence of 10mM H_2_O_2_ (ε_240nm_ = 39,40 M^−1^ cm^−1^).

Protein concentration was determined according to the Bradford method (1976) [48] using the Protein Assay Kit (BioRad). Medium inoculated with *B. adusta* CCBAS 930, but without the addition of anthracyclines served as control in all enzymatic activity assays.

### 4.5. Determination of Phenolic Compounds (PhC) and Free Radical (SOR) Contents

The content of PhC was determined spectrophotometrically at A_740nm_ according to Singleton and Rossi (1965) [49] with a slight modification [50]. SOR levels were estimated based on the detection of superoxide-induced formazan formation from nitrotetrazolium blue (NBT) (A_560nm_) [51]. Untreated medium with DNR or DOX at a concentration of 10 µg/mL was used as control.

### 4.6. Determiantion of Antioxidative Activity of Initial Antracycline Solutions and Post-Culture Fluids of B. adusta CCBAS 930

Antioxidant activity was measured using the DPPH^•^ radical scavenging assay according to Brand-Williams et al. (1995) [52]. Each 100 μL of post-culture fluids and initial DNR and DOX (10 µg/mL) solutions were mixed with 100 μL of 25 mM DPPH^•^ solution in 96% ethanol. Following 30 min incubation at room temperature, sample absorbance was measured (A_515nm_) using 96% ethanol as a blank sample.

### 4.7. Determination of pH of Post-Culture Fluids

The pH of post-culture liquids was measured periodically using potentiometric methods.

### 4.8. Phytotoxicity Assay

Phytotoxkit (Tigret, Poland) was used to determine the direct effects of the untreated and treated DNR and DOX on ermination index (GI) and root growth inhibition (RGI) of seeds of *Lepidium sativum* in comparison to controls (distilled water) in a reference soil.

### 4.9. Ecotoxicity Assay

The Microtox^®^ (Model 500 Analyzer, London, UK) toxicity assay was used to evaluate the inhibition of luminescence of *V. fischeri* marine bacteria for untreated medium supplemented with DNR and DOX (10 µg/mL) and post-culture liquids of *B. adusta* CCBAS 930 according to the manufacturer’s protocol.

### 4.10. Genotoxicity Assay

The genotoxicity assay was performed using SOS ChromoTest (distribution Tigret, Poland) according to the manufacturer’s protocol. Moreover, the S9 fraction (lyophilized rat liver) was used to estimate the promutagenic potential of tested compounds before and after treatment with *B. adusta* CCBAS 930. Briefly, overnight bacteria cultures were grown in fresh LB medium to an optical density (OD_600nm_) of 0.5–0.6, diluted 10-fold in double strength LB medium (20 g tryptone/L, 10 g yeast extract/L, 20 g sodium chloride/L, pH 7.4) and mixed (*v/v*) with the tested compounds, e.g., potential mutagens (or promutagens) and solvents. A negative control (distilled water) was always included in each assay. The bacteria were exposed to different initial concentrations of DOX and DNR and post-liquid cultures of *B. adusta* CCBAS 930 after anthracycline treatments and cultured for 1.5 h at 37 °C. β-galactosidase (β-gal) and alkaline phosphatase (AP) were assayed in 96-well plates. Significant genotoxic activity was defined as an adjusted induction factor (CIF) equal or greater than 1.2.

### 4.11. Cell Culture with Conditioned Medium

The human skin fibroblasts cell line BJ (ATCC CRL-2522) was obtained from American Type Culture Collection (ATCC, distributors: LGC Standards, Łomianki, Poland). BJ cells were maintained in MEM medium without phenol red supplemented with 10% fetal bovine serum (FBS). Cells were maintained at 37 °C in a humidified atmosphere with 5% CO_2_. Cells were seeded in 96-well culture plates (Costar, St. Louis, MO, USA) at a density of 5 × 10^3^ per well and initially cultured before the experiment for 24 h. Subsequently, the medium was changed to a fresh one with an equal amount of conditioned medium (Control 1—cell culture medium with PBS, Control 2—cell culture medium with Park and Robinson medium for B. adusta CCBAS 930 culture for fungi culture, DOX—cell culture medium with doxorubicin, DOX metabolite—cell culture medium with doxorubicin after fungi culture, DNR—cell culture medium with daunomycin, DNR metabolite—cell culture medium with daunomycin after fungi culture) which was always 10% of the total cell culture medium.

### 4.12. Resazurin Reduction Assay

The resazurin reduction cell viability and metabolism assay was performed according to a previously described method [53]. On the day of analysis, a working solution of 60 µM resazurin was prepared in medium containing 1% FBS. After 24 or 48 h of treatment, cells with studied conditioned media, medium in the wells were replaced on a working solution of resazurin (100 µL) and the plates were placed in 37 °C. Fluorescence was measured with an excitation wavelength of 530 nm and emission 590 nm on a FilterMax F5 Multi-Mode microplate reader (Molecular Devices, Corp., Sunnyvale, CA, USA) for 1 and 2 h after dye addition.

### 4.13. Measurement Reactive Oxygen Species (ROS) Production

The ROS measurement was performed according to a previously described method [54]. To determine the ability conditioned medium (Control 1, Control 2, DOX, DOX metabolite, DNR, DNR metabolite) to induce ROS production in the cells, 5 μM H2DCFDA was applied. The cells were incubated in H_2_DCFDA in serum-free and phenol red-free medium for 45 min before conditioned medium treatment. After 24 and 48 h of incubation of the cells with increasing concentrations of compounds (5% CO_2_ at 37 °C), the culture medium was replaced with fresh medium to remove extracellular residual DCF and studied compounds to reduce the fluorescence background. Cells treated with 0.3% hydrogen peroxide (H_2_O_2_) were used as a positive control (result not shown). DCF fluorescence was measured using a microplate reader (FilterMax F5) at maximum excitation and emission spectra of 485 nm and 535 nm, respectively.

### 4.14. Caspase-3 Activity

Caspase-3 activity was assessed according to previous described method Nicholson et al. [55] After 24 or 48 h of treatment with studied media culture plates were frozen in −80 °C and kept until assay. Cells were lysed using (frozen procedure) and lysis buffer (50 mM HEPES, pH 7.4, 100 mM NaCl, 0.1% CHAPS, 1 mM EDTA, 10% glycerol, and 10 mM DTT) at 10 °C for 10 min. The lysates were incubated with substrate Ac-DEVD-pNA for caspase-3 at 37 °C. Cells treated with 1 μM staurosporine were used as a positive control (results not shown). After 30 min, absorbance of the lysates at 405 nm was measured using a microplate reader (FilterMax F5 Multi-Mode microplate reader). The amount of colorimetric product was continuously monitored for 120 min. The data were analyzed using Multi-Mode Analysis software (Molecular Devices, Corp., Sunnyvale, CA, USA).

### 4.15. Calcein AM Staining

Calcein AM staining was performed to measure intracellular esterase activity in neocortical cell cultures 24 and 48 h after treatment with Control 1, Control 2, DOX, DOX metabolite, DNR and DNR metabolite diluted in MEM medium containing 10% FBS using a fluorescence microscope (LSM 700, ZEISS) [20].

### 4.16. Statistical Analysis

Data are presented as means ± standard deviation (SD) of three (biochemical analysis) and six (cell cultures and cytotoxicity assays) independent experiments. Data were analyzed using one-way analysis of variance (ANOVA) followed by Tukey’s multiple comparison procedure and Principal Component Analysis (PCA) (normalized VARIMAX and Kaiser criterion) [56] using STATISTICA v10.0 Software (StatSoft Poland)

## 5. Conclusions

In conclusion, it should be emphasized that the *B. adusta* strain CCBAS 930 exhibited biotransformation activity towards anthracycline antibiotics such as DNR and DOX. This activity was mainly related to the production of extracellular HRP-like and VP peroxidases. Our study showed that DNR and DOX were toxic for *Vibrio fischeri* bacteria, *L. sativum* L. seeds and BJ skin fibroblasts cell line before biotransformation. Our study suggested a ROS-dependent cytotoxicity mechanism associated with caspase-3 activity. Biotransformation products, e.g., phenols can protect fibroblast cells by ROS during biological treatment of anthracyclines in *B. adusta* CCBAS 930 cultures. Moreover, phenols formed during anthracycline biotransformations showed antioxidant activity. It can be assumed that not only oxidoreductases, but also phenolic compounds formed during biotransformation of anthraquinone derivatives were responsible for the detoxification of these compounds. Based on these results, further studies require the characterization of the main VP peroxidase involved in anthracycline bioremoval. Since no phytotoxicity after AQA treatment with *B. adusta* CCBAS 930 was recorded, it is also planned to conduct a pilot study on the use of post-culture liquids for agricultural irrigation after anthracycline removal.

## Data Availability

The data presented in this study are available on request from the corresponding author.

## Sample Availability

Samples of the compounds are not available from the authors.

## Appendix A

**Table A1:** Genotoxicity levels of DNR and DOX samples before and after treatment by *B. adusta* CCBAS in 20-days stationary cultures.

Samples	C	V	CIF	SD
DNR before treatment (initial solution 10 µg mL^−1^)	10	-	**13.42**	0.54
	5		**5.68**	0.11
	2.50		**2.80**	0.08
	1.25		**1.54**	0.06
	0.62		**1.21**	0.08
	0.31		**1.60**	0.03
	0.15		**2.50**	0.06
DOX before treatment (initial solution 10 µg mL^−1^)	10	-	9.58	0.12
	5		**3.70**	0.08
	2.50		**3.27**	0.08
	1.25		**2.53**	0.02
	0.62		**1.23**	0.04
	0.31		**1.21**	0.08
	0.15		**2.88**	0.10
DNR after treatment by *B. adusta* CCBAS 930	-	100	0.98	0.02
		50	0.46	0.05
		25	0.73	0.06
		12.50	0.11	0.02
		6.25	0.40	0.04
		3.12	0.93	0.04
		1.56	0.72	0.08
DOX after treatment by *B. adusta* CCBAS 930	-	100	1.13	0.14
		50	1.08	0.06
		25	0.65	0.06
		12.50	0.98	0.07
		6.25	1.01	0.09
		3.12	0.99	0.06
		1.56	1.06	0.06

C—concentration in µg mL^−1^; V—in %, CIF—corrected induction factor, SD—standard deviation, genotoxic samples are indicated in bold.
